# Physical activity level and factors associated with perceived stress among Peruvian university professors during the COVID-19 pandemic

**DOI:** 10.1016/j.heliyon.2023.e16439

**Published:** 2023-05-23

**Authors:** Liliana Cruz-Ausejo, J. Osada, L. Rueda-Torres, Nataly Briggete Ingunza Lastra, Miguel Alfredo Carrasco-Muñoz, Victor Juan Vera-Ponce

**Affiliations:** a Facultad de Ciencias de la salud, Universidad Científica del Sur, Lima 15074, Peru; b Universidad Peruana Cayetano Heredia, Lima 15012, Peru; c Universidad Nacional Daniel Alomia Robles, Huánuco 10001, Peru; d Universidad Nacional Hermilio Valdizan, Huánuco 10160, Peru; e Instituto de Investigación em Ciencias Biomédicas, Universidad Ricardo Palma, Lima 150140, Peru; f Universidad Tecnológica del Perú, Lima 15046, Peru

**Keywords:** Physical activity, Psychological stress, Occupational health, Mental health, Coronavirus, Faculty (MeSH terms)

## Abstract

**Introduction:**

The COVID-19 pandemic led to the transition to remote work, triggering variations in stress and physical activity (PA), associated with context-specific instability.

**Objective:**

To identify the association between perceived stress (PS) and the level of physical activity (PA) and explore its relationship with the sociodemographic, family, work and individual characteristics of professors working remotely during the COVID-19 pandemic.

**Material and methods:**

Cross-sectional analytical study based on a virtual survey of professors. PS was assessed using the Perceived Stress Scale (PSS-14), and PA using an International Physical Activity Questionnaire. The prevalence of high PS and the association with PA were estimated using a Poisson regression analysis with robust variance that estimated crude prevalence ratios (cPR) and adjusted prevalence ratios (aPR) with a 95% confidence interval (CI). Five models were developed to assess associations of PS and PA with sociodemographic, family, work, and individual variables.

**Results:**

The information of 191 professors was analyzed; 39.27% were women, aged 52 (41–60). The prevalence of high stress was 47.12%. The age and being head of household did not show significant individual associations with PS. However, the regression analysis assessing the association of PS and other factors showed that compared to the moderate PA group, a statistically significant association was found between stress and high PA (aPR = 0.19; 0.06–0.59), low PA (aPR = 1.43; 1.02–2.01), mainly influenced by age, being head of household and sleep quality.

**Conclusion:**

Stress was associated with PA level, family and individual factors. These findings allow identifying characteristics, such as being head of household, age and quality of sleep among teachers, as being associated with a higher probability of having high stress. Subsequent studies should consider the role of individuals and working conditions as part of occupational health surveillance, given the presence of hybrid education in the education sector.

## Highlights

• University professors with a LPA are nearly 45% more likely to have high levels of EP compared to those with MPA.
• The fact of being heads of households and performing LPA increases the probability of having high levels of stress by up to 58%.
• Future studies should consider the role of family and individual factors, as part of occupational surveillance.

## 1 Introduction

Mental health conditions impact the world economy, generating a high cost at the social level and on labor organizations. In the United Kingdom, it is estimated that 213 300 days of work are lost every year as a result of anxiety, depression and stress [1]. In the teaching population, stress represents a psychosocial risk conditioned by several factors, such as workload, physical activity [2,3], lack of support [4], interpersonal relationships, personal life, poor sleep quality [3,5,6], culture [7]; Added to the social structure of working context of the profesors. Thus is relevant to comprehend the factors that can modify their physical and mental status.

From a sociological perspective, the Cultural Capital theory by Bordieu [8] describes the existence of symbolic capital as a disputable possession within a social group. In the case of professors, this wealth justifies the maintenance of status and power through its several forms: incorporated (knowledge), objectified (cultural goods) or institutionalized (capacity of the group to accredit knowledge). These Capital forms acquire great relevance to their motivation and struggles to the seeking for a status and habitus superior to others which creates an environment of constant competition.

From an occupational point of view, in Perú in 2020, the professors hired hourly or part-time integrated 58.6% of the teaching workforce [9]. Usually, these professors spend their work hours in classroom activities; that is some teachers did not receive a compensatory salary for hours dedicated to the development of educational material and tuition, among other tasks [9,10]. Similarly, the regime of a full-time professor and exclusive dedication combines teaching, administrative and scientific research activities nonetheless, the salary in this group included the non-schooled hours [9]. In both cases, the aforementioned activities are though to evaluate with an instrument given the multiplicity of tasks they perform [10]. Moreover, this particular feature of the teacher labor regimen allows professors to work in different institutions in order to increase their economic income, despite the work overload.

Furthermore, conditions such as upbringing and the role of being the head of the family which implies the burden of home activities that can overlap with work must be added [11]. Especially among parents of children of scholarly age or economically dependent, the raises of stress and post-traumatic stress symptomatology have emerged as a consequence of work-life imbalance and perceived financial instability [12]. In addition, current conditions such as the accelerated transition to remote work (RW) [2,11,13] and time spent on it, over 9 months per year in university education on average higher workload with a ratio of 36.5 students per full-time university professor in the Peruvian highlands [9] have exacerbated a hostile environment for their work with implications not only on the perception of stress but also on the practice of physical activity (PA) [14,15].

PA is widely recognized as a protective factor against comorbidities, such as diabetes, hypertension [16], and especially stress [17]. During the pandemic its practice was recommended, however, according to Bogaert et al. [18] sports activity and no other type of physical exercise performed during free time, prevent stress; that is, not all exercise performed during occupational work is beneficial. Even though, the practice of PA has been highly-encouraged among people to prevent or mitigate negative effects on mental health [19], recent reports have shown that work (overload in RW) [20], and individual conditions such as poor quality of sleep [7,20] within the context of COVID-19 are linked to a significant decrease in mobility, walking and an increase in sedentary lifestyle [21]. And since the lockdown has restricted some PA options, a decrease in this practice and a non-positive impact on stress might be expected.

Therefore, this study was aimed at identifying the association between perceived stress (PS) in the last month of work and the level of PA among university professors who worked remotely within the context of the COVID-19 pandemic. Likewise, we explored the relationship of this association with sociodemographic, familiar, work and individual characteristics.

## 2 Materials and methods

### 2.1 Participants and study design

This was a cross-sectional study based on a virtual survey. We assessed the PS and the level of PA in university professors who maintained a current employment relationship to date and worked for at least one month in the RW modality, at a public university located in the department of Huánuco, Peru during the period of March–June 2021. In this period, the population was still under lockdown, with social restrictions and university professors carrying out RW.

### 2.2 Definition of variables

#### 2.2.1 Perceived stress

The variable of interest was PS in the last month. This was measured using the Cohen Perceived Stress Scale (PSS-14) [22], validated in the Spanish-speaking population [23], more specifically in university teachers with good psychometric properties as ɑ = 0.82 (0.76-0.87; confidence interval [CI] 95%) [4]. The PSS-14 has 14 questions that assess two dimensions of stress: “perception of stress in daily situations” (negative dimension: questions 1,2,3,8,11,12,14), which addresses negative experiences and situations, and “stress control” (positive dimension: questions 4,5,6,7,9,10,13). Each item receives a Likert-type score from 0 “never” to 4 “very often”. The total score varies between 0 and 56 points, with a higher score reflecting a greater presence of stress and vice versa [22,24]. Currently, there is no consensus on the cut-off point for the level of stress, and thus, the authors established categories by tertiles or their means [[25], [26], [27], [28]]The median of stress considered 24 as the cut-off point to establish low and high levels of stress.

#### 2.2.2 Physical activity

This independent variable was assessed with the 7-item short physical activity questionnaire (International Physical Activity Questionnaire-IPAQ), which has been translated and adapted into Spanish and in more than 12 countries with good psychometric properties reliability r = 0.76 (95% CI, 0.73–0.77) [29]. In Peru it has been used as part of the surveillance of Nutrional status in population (VIANEV, in spanish). The short version assesses three types of daily activities of moderate intensity, vigorous intensity, and walking. The score is calculated by multiplying the basal metabolic rate (metabolic equity of task - MET) 3.3; 4.0; and 8.0 METs, respectively for the minutes/week of activity. The total result is obtained from the sum of these activities according to the algorithm detailed in *Guidelines for data Processing and Analysis of the International Physical Activity Questionnaire (IPAQ)-Short Form* [30].

#### 2.2.3 Confounding variables

The confounding variables were identifed using a directed acyclic graph [[31], [32]]: Sex (male, female), head of household (yes, no), age, months in remote work, working hours per week (<30 h/week, ≥30 h/week), dedication (partial or full-time), and quality of sleep. This last measure determined by the Pittsburgh Sleep Quality Questionnaire, of Buysee [33], consists of 19 questions and the result is distributed into four categories. A score less than 5 indicates “no sleep problems”, from 5 to 7 “deserves medical attention”, from 8 to 14 “deserves medical attention and treatment” and from 15 points to more “serious sleep problems”.

### 2.3 Procedure and data collection

The questionnaire was designed on the Typeforms platform (https://www.typeform.com/es/) and was distributed to professors by email. To reduce selection bias due to overreporting, responses were filtered by Network ID in order to avoid duplicate responses. To increase the response rate, participation reminders were sent every 2 weeks for 4 months. The data were exported to an Excel spreadsheet where two researchers LCA, and LRT, independently cleaned the data. There were no lost data as to fill out the survey it was necessary to complete the previous question.

### 2.4 Sample size

The sample size was determined with the STATAv.15 program. According to the literature, a difference of 28.1% in the proportions of stress between those with high PA and low PA (20.4% and 48.5%) [34] and a ratio of 1 was assumed. Thus, considering a 95% CI and statistical power of 80%, a minimum sample size of 102 participants was calculated.

Additionally, the entry of 8 confounding variables in the multiple regression model was considered, for which it was necessary to have a minimum number of participants. This was estimated using the formula: *N ≥ 50 + 8 m* (where “m” is the number of independent variables) [35] and thus, the minimum number of subjects required was 114. Ultimately, 191 participants filled out the survey.

### 2.5 Statistical analysis

Since the quantitative variables did not meet the normality principle, they were summarized by their median and interquartile range (IQR), while the qualitative variables were represented by absolute and relative frequencies. Chi-square, Fisher's exact and U-Mann Whitney tests were applied to compare the low and high-stress groups, provided the covariates were categorical or quantitative, respectively. Multiple regression analysis was performed for PS, PA, and covariates. Multivariate analysis used a Poisson-family generalized linear model, log link function with robust variance [36,37] in the STATA 15 program (STATA Corp, College Station, TX, US). Four models were developed, based on the statistical strategy of Shaffer et al. [38] to assess unique associations to which variables were added as follows, model 1: unique associations of sociodemographic factors (age, gender) were tested, model 2: family variables (head of household and economically dependent children), model 3: working factors (months in remote work, professional dedication, working hours per week) and model 4: individual factors (sleep quality). Each model assessed unique associations of the factors with PS and PA. Finally, a model that incorporated all the previous variables was developed. A p-value <0.05 was considered statistically significant.

### 2.6 Ethical considerations

This study was conducted according to the guidelines of the “Declaration of Helsinki”, and the procedures were approved by the Research and Institutional Ethics Committee of the Universidad Científica del Sur (UCSUR), Lima, Peru in Certificate No.299-CIEI-CIENTIFICA-2020. Accepting the (virtual) informed consent was required to continue filling out the survey.

## 3 Results

### 3.1 Characteristics of the study population

Information was collected from 191 participants, of whom 39.27% were women, the median age was 52 (41–60) years old, and the prevalence of low PA was almost double that of high PA. The Sociodemographic, family, work and personal characteristics of the participants are shown in Table 1.

**Table 1:** General characteristics of the participants (n = 191).

Characteristics			
	n	%	
***Socio-demographic characteristics***			
Gender			
Male	116	60.73	
Female	75	39.27	
Age^a^			52 (41–60)
***Family characteristics***			
Head of household			
Yes	156	81.68	
No	35	18.32	
Economically dependent children			
Have no children	33	17.28	
Have no economically dependent children	39	20.42	
Have economically dependent children	119	62.30	
***Working characteristics***			
Months doing remote-work^a^			10 (8–12)
Working hours per week			
< 30 h/week	75	39.27	
≥ 30 h/week	116	60.73	
University dedication			
Part-time basis	59	30.89	
Full-time basis	132	69.11	
***Individual Characteristics***			
Quality of sleep			
No sleep problems	29	15.18	
Deserves medical attention	57	29.84	
Deserves attention and medical treatment	97	50.79	
Severe sleep problems	8	4.19	
Perceived stress			
Low-perceived stress	101	52.88	
High-perceived stress	90	47.12	
Physical activity			
LPA^b^	93	48.69	
MPA^b^, ^c^	42	21.99	
HPA^d^	56	29.32	

a Median and interquartile range.

b Low physical activity.

c Moderate physical activity.

d High physical activity.

### 3.2 Perceived stress and physical activity

**Fig. 1:**
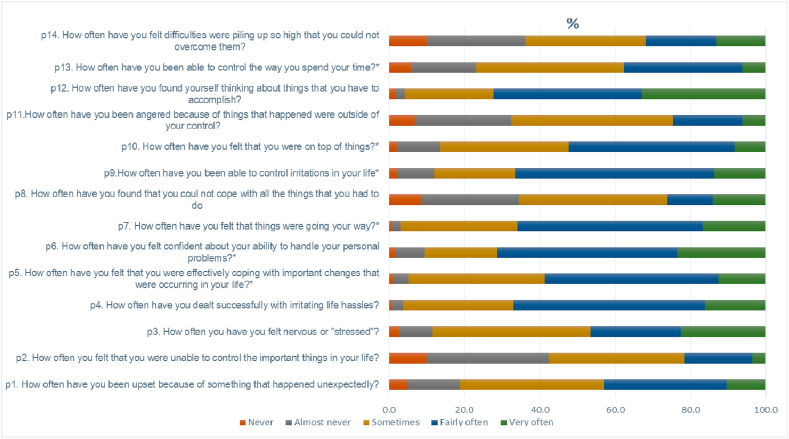
Distribution of responses for the Perceived stress scale-PSS-14.

The median for low stress was 19 (IQR:15–22, range 4–24) and 32 (IQR: 28–38, range: 25–50) for high stress. The characteristics of the population according to stress levels are summarized in Table 2 we observed that only working hours per week, quality of sleep and physical activity showed statistically significant association with PS. Moreover, Fig. 1 shows the distribution of responses for the 14 items of PS. The first dimension “perception in stressful situations” (questions: 1,2,3,8,11,12,14), is assessed by questions n°1 *How often have you been affected by something that has happened unexpectedly?* and n°12 *How often have you thought about the things you have left to do?*, 32.4% and 39.26% of professors said they often felt affected. While in the second dimension “subjective control” (questions: 4,5,6,7,9,10,13), assessed by question n°6 *How often have you been confident about your ability to handle your personal problems?* 47.64% of professors stated that they felt confident.

**Table 2:** Bivariate analysis of perceived stress in the last month (N = 191).

	Perceived stress			%	p value
Characteristics	Low	%	High		
	n = 101		n = 90		
	n		n		
**Socio-demographic characteristics**					
Gender^b^					
Male	62	53.45	54	46.55	0.845
Female	39	52.00	36	48.00	
Age^c^	51 (41–60)^a^		52 (43–60)^a^		0.955
**Family characteristics**					
Head of household^b^					
Yes	81	51.92	75	48.08	0.576
No	20	57.14	15	42.86	
Economically dependent children^b^					
Have no children	17	51.52	16	48.48	0.694
Have no economically dependent children	23	58.97	16	41.03	
Have economically dependent children	61	51.26	58	48.74	
**Working characteristics**					
Months doing remote-work^c^	10 (8–12)^a^		10 (8–12)^a^		0.854
Working hours per week^b^					
< 30 h/week	50	66.67	25	33.33	**0.002**
≥ 30 h/week	51	43.97	65	56.03	
University dedication^b^					
Part-time basis	36	61.02	23	38.98	0.132
Full-time basis	65	49.24	67	50.76	
**Individual characteristics**					
Quality of sleep^d^					
No sleep problems	28	96.55	1	3.45	**<** 0.001
Deserves medical attention	43	75.44	14	24.56	
Deserves attention and medical treat.	29	29.90	68	70.10	
Severe sleep problems	1	12.50	7	87.50	
Physical activity^b^					
LPA^e^	24	25.81	69	74.19	**< 0.001**
MPA^f^	24	57.14	18	42.86	
HPA^g^	53	94.64	3	5.36	

a Median and interquartile range.

b Chi-square test.

c Mann Whitney *U* test.

d Fisher's exact test.

e Low physical activity.

f Moderate physical activity.

g High physical activity.

### 3.3 Factors associated with perceived stress and physical activity

The results of the regression model that assessed the independent associations among sociodemographic, family, work and individual factors with PS and PA are shown in Table 3. Models 1 and 2 did not show significant associations for sociodemographic and family characteristics with PA and PS. While model 3 identified that professors who worked more than 30 h/week presented a 50% higher probability of presenting high stress in comparison to those working less than 30/h week and when PA remained constant. Model 4, showed that those with poorer sleep quality (deserve medical attention and deserve attention plus medical treatment) present from 94% to 98% higher probability of having high stress, compared to those who just deserve medical attention and adjusted for PA.

**Table 3:** Multivariate regression analysis of perceived stress and influencing factors.

Perceived stress	cPR	(95% CI)	p	Model 1			Model 2			Model 3			Model 4			Model 5		
				aPR	(95% CI)	p	aPR	(95% CI)	p	aPR	(95% CI)	p	aPR	(95% CI)	p	aPR	(95% CI)	p
Physical activity																		
HPA	0.13	(0.04–0.40)	<0.001	0.12	(0.04–0.40)	**<0.001**	0.13	(0.04–0.41)	**0.001**	0.12	(0.04–0.39)	**<0.001**	0.19	(0.06–0.61)	**0.005**	0.19	(0.06–0.59)	**0.004**
MPA	ref.			ref.			ref.			ref.			ref.			ref.		
LPA	1.73	(1.20–2.51)	0.004	1.74	(1.20–2.52)	**0.003**	1.78	(1.23–2.58)	**0.002**	1.64	(1.14–2.35)	**0.008**	1.47	(1.03–2.09)	**0.032**	1.43	(1.02–2.01)	**0.038**
Age	1.00	(0.99–1.01)	0.956	1.00	(0.99–1.01)	0.511										0.98	(0.97–0.99)	**0.026**
Genre																		
Male	ref.			ref.												ref.		
Female	1.03	(0.76–1.40)	0.845	0.92	(0.71–1.19)	0.533										0.94	(0.75–1.19)	0.626
Head of household																		
No	ref.						ref.									ref.		
Yes	1.12	(0.74–1.70)	0.589				1.27	(0.86–1.87)	0.225							1.58	(1.05–2.36)	**0.027**
Economically dependent children																		
HNCh	ref.						ref.									ref.		
HNEDCh	0.85	(0.50–1.42)	0.526				0.88	(0.58–1.34)	0.562							0.91	(0.59–1.41)	0.670
HEDCh.	1.01	(0.68–1.50)	0.979				0.84	(0.59–1.21)	0.349							0.82	(0.60–1.14)	0.245
Months doing remote work	1.00	(0.94–1.05)	0.916							0.98	(0.94–1.03)	0.480				0.98	(0.93–1.03)	0.370
Dedication																		
Partial-time basis	ref.									ref.						ref.		
Full-time basis	1.30	(0.91–1.87)	0.153							0.96	(0.69–1.34)	0.803				0.95	(0.63–1.44)	0.824
Working hours per week (h/w)																		
<30 h/week	ref.									ref.						ref.		
≥30 h/week	1.68	(1.17-2-41)	0.005							1.50	(1.05–2.13)	**0.025**				1.48	(0.98–2.22)	0.060
Quality of sleep																		
No sleep problems	0.14	(0.02–1.02)	0.052										0.23	(0.03–1.54)	0.128	0.24	(0.04–1.59)	0.140
Deserve medical attention	ref.												ref.			ref.		
Deserve at. and medical treat	2.85	(1.78–4.59)	<0.001										1.94	(1.24–3.04)	**0.004**	1.92	(1.24–2.95)	**0.003**
Severe sleep problem	3.56	(2.10–6.03)	<0.001										1.98	(1.19–3.30)	**0.009**	2.61	(1.51–4.53)	**0.001**
AIC initial				1.662			1.662			1.662			1.662			1.662		
AIC final				1.461			1.469			1.459			1.404			1.468		

p < 0.05 significative value, **cPR**: crude Prevalence ratio, **aPR** adjusted Prevalence Ratio, **HNCh**: have no children, **HNEDCh**: have no economically dependent children, **HEDCh**: have economically dependent children, **HPA:** high physical activity, **MPA**: moderate physical activity, **LPA**: low physical activity.

Model 5, adjusted for all the above variables, showed the association between the level of PA and PS. The level of high PA was shown to be a protective factor against high stress when the confounders remained constant. In addition, among professors who are heads of households, the probability of having high stress increased by almost 60% compared to those who are not heads of households, after adjustment for confounders. Age was identified as a protective factor for high stress. For each year of age, this probability was reduced by 2%, after adjustment for sociodemographic, family and individual variables.

Additionally, requiring medical attention plus treatment and having serious sleep problems almost doubled and tripled the probability of high PS compared to those who only required medical attention.

## 4 Discussion

### 4.1 Main findings

Our results show that about half of the participants had a low PA level, with more than 74% of professors presenting high stress. The presentation of high stress was associated with a low level of PA among university professors, after adjustment for sociodemographic, family, work and individual variables.

### 4.2 Comparison with previous studies

The participants in this study were predominantly male, similar to the national proportion of university professors [39], and other studies [40,41] but different from the studies by Leire et al., Freitas et al. and Camargo et al., in which women exceeded 50% of the study participants. The median age was around 52 years old, which contrasts with the national average age of professors [39] and other studies describing the age as being around the 4th decade of life [15,[41], [42], [43]]. These results are expected and partly explained by the current requirements of university teaching, requiring greater experience and trajectory, which are characteristics acquired over time, especially among professors of public universities [9].

Our study showed a higher figure of professors in full-time dedication than that reported in the 2nd National University Census [44]. This difference is probably due to the response options of the questionnaire, which did not include the *exclusive dedication* option, thereby not allowing distinguishing the latter from full-time work. However in terms of workload the exclusive and full-time modalities are considered equivalent [9]. In reference to the weekly working hours, the difference was greater, with more than 60% working more than 30 h/week versus the 18 h average schooled-hours of teaching load by Peruvian university professors in the Highlands [9]. We have to considered that this figure did not include non-schooled hours, it meant that this difference could be attributed to extra-worked hours (educational material, design of educative test, etc.) and mainly non-paid back hours; nonetheless, this percentage is similar to the average reported in Brazilian professors [42,43].

The context of the pandemic and RW in an atypical environment [13] have led to a set of greater workload [2] with the resulting increase in anxiety, depression and stress symptomatology [45]. In this sense, the perception of high stress in this study varied compared to other reports [[45], [46], [47]]. On one hand, it is lower than that reported worldwide [45] and nationwide [46], which exceeds 60% while being significantly higher than that experienced by teachers of Paraguayan education [47]. Whereas it is similar to the reports of the Polish teaching population of elementary education [48] and Brazilian university professors [40]. These differences could be justified by the temporal disparity of the reports before and during different stages of the pandemic, in Huánuco the second wave was officially stated in 31st January inducing to mandatory isolation [40,43,45,48] and indirectly extending the RW in education. In addition to the several factors as the representativeness of the population [46], cultural context, and educational level, which influence the perception of work stress [7].

Preview literature recognizes the value and impact of PA on health [49]. Although the levels of low and high PA found in this study are worrying, the results are consistent with the insufficiently active profile of university professors, even before the pandemic [42,43,50] while, at the same time, being better than that of inhabitants of the Peruvian highlands in which low and high PA levels reach 66.2% and 5.6%, respectively [51]. In addition, if we compare the proportion of professors with high PA to that of professors from Lima (37%) [52], there are discrepancies possibly attributed to the requirement of the level of teaching and administrative load [53], characteristics that affect the availability of free time to carry out personal life activities [18,48].

In relation to sociodemographic characteristic, the findings of this study suggest that both low and high PA are directly and inversely associated with the presence of high stress even adjustment for characteristics such as age, which had a protective role in professors with a good level of PA unlike to Portuguese professors who show a tendency to increase stress with respect to age [41]. A reduction in PS in relation to age and the practice of PA in professors could be linked to years of teaching experience; that is, those with a longer service time have a better performance, knowledge of educational strategies, a reflection of professional maturity and experience which, added to the appropriate management of time and responsibilities, would allow the inclusion of leisure activities outside work [18].

The relationship between gender, stress, and PA has been extensively studied, showing that women tend to have higher stress levels and poor PA [50]. This relationship is maintained in professors [[41], [42], [43]] and is associated with concern for the fulfillment of work, and personal life, specifically in the performance of household chores [54] and maternal work [11]. However, found no differences in stress levels and PA according to sex, which could be explained by shared parenting responsibilities, with greater partner support at home, which translates into a reduction in the gap between family and work obligations, reducing the family burden on women [43].

In addition, familiar factors as house demands a greater commitment for the head of household, who bears most of the economic burden, and thus, it is expected that those who assume this role will experience greater physical and mental wear, associated with the presence of children, economically dependents (elderly or people with disability) and even the reduction or little PA practice in non-working hours [40]. This suggests that the differences observed for PS and PA in the heads/non-heads of households could be related to the role of the person in charge of the household, rather than gender *per se*.

Additionally, more than 84% of those surveyed presented some problem related to sleep quality, requiring attention and/or medical treatment, being well above that reported by López-García et al., Crepaldi et al. and Freitas et al. [5,6,47]. Preliminary reports suggest that professors present sleep problems, such as daytime sleepiness [55] and insomnia [56], an issue that has been aggravated by the transition to RW, reduction in financial stability [13,57] and PA practice [57] due to confinement, which obliges the professors to take several jobs, extending their working hours and workload, with the consequent affectation of their quality of life [55,56] and perceived stress [6].

Moreover, it is necessary to mention that the results observed in this study are likely to be influenced by the coincidence in information collection and the start of the 2nd wave of the pandemic in the department of Huanuco. This can be coupled with other factors related to the organization of work [58], instability contextual and truncated expectations as the main source of anxiety, stress [4], and alteration of sleep quality [5,6] which altogether deteriorate the mental health status of professors.

Finally, by the time of this publication the current period of COVID-19 is basically past all over the world, notwithstanding this research sustains the importance of maintaining healthy PA levels even under though contexts which has a positive impact on the perception of stress in professors. It should be taken into account that the acceleration of the digital transformation to improve teaching processes, mentioned in the *“National Policy for Higher Technical-Productive Education”* by 2030 [59], indicates the continuity of blended and distance learning in addition to the implementation of telecommuting set by peruvian government which ensures the permanence of this labor modality. Consequently, adopting a culture of promotion and prevention of health through assistance and education programs for professors, as well as having policies to promote the extension of occupational health to telecommuting, are necessary to reduce psychosocial risks in this population.

### 4.3 Strengths and limitations

We can identify four limitations. First, due to the cross-sectional design of the study in which we only estimated association and not causality, the interpretation of the results must be taken with caution. Second, the sample included may not be representative of university professors in our country, since the participants belonged to a public entity, and thus, we cannot extrapolate the results to private universities in another locality. Third, the data collection took place during the months of March–June 2021, in which inhabitants were still under lockdown, and there was an increase in COVID-19 cases in Peru, which could have influenced the responses, given the adverse context. Therefore the effect of the stated association may not just be attributed to PS but rather also to COVID-19. And fourth, reverse causality problems could arise since there is a temporal difference and recall bias in the nature of the responses: stress, compared to the last month of work and PA in the last week. However, other studies support the direction of the association presented [34].

On the other hand, this is the first study among Peruvian rural professors that explores the relationship between PS and PA and other factors during the COVID-19 pandemic. This information may help to design specific measures to prevent high-level stress and deterioration of PA during RW and hybrid education which are considered as being permanent in higher education. Additionally, the occupational health among Peruvian teachers, in general, is one of the most relegated, there are no reports or evidence of the state of workers during this context, and thus, this study provides a first look at professor status and conditions during the pandemic.

## 5 Conclusion

The results support the association between the level of PA and PS in university professors who do RW within the context of the COVID-19 pandemic. Those presenting a high PA level were less likely to have high stress unlike those with low PA adjusted for sociodemographic, work and individual characteristics, with age, being head of household and having sleep problems being determining factors.Furthermore, the reality of public universities in low-and middle-income countries is marked by a lack of infrastructure, technological equipment, multimedia platforms for RW and therefore, future studies must consider the influence of organizational, labor and support factors for Peruvian highland professors with similar features in the context of hybrid education or telecommuting in addition to ensuring the implementation of occupational health surveillance programs in this sector.

## Author contribution statement

Liliana Cruz-Ausejo: Conceived and designed the experiments; Performed the experiments; Analyzed and interpreted the data; Contributed reagents, materials, analysis tools or data; Wrote the paper.

Jorge Osada: Conceived and designed the experiments; Contributed reagents, materials, analysis tools or data; Wrote the paper.

Lenin Rueda -Torres: Performed the experiments; Analyzed and interpreted the data; Contributed reagents, materials, analysis tools or data; Wrote the paper.

Nataly Briggete Ingunza Lastra; Miguel Carrasco Muñoz; Victor Juan Vera-Ponce: Analyzed and interpreted the data; Contributed reagents, materials, analysis tools or data; Wrote the paper.

All authors approved the final version of this manuscript.

## Funding statement

This work was supported by Universidad Científica del Sur (PE) [“Fondo Beca Cabieses 2020-2”].

## Data availability statement

Data associated with this study has been deposited at Mendeley Data: Cruz-Ausejo, Liliana (2022), “Physical activity vs perceived stress/teachers/2021”, Mendeley Data, V1, https://doi.org/10.17632/4ng5pnrcn3.1.

## Declaration of competing interest

The authors declare the following financial interests/personal relationships which may be considered as potential competing interests: Liliana Cruz-Ausejo reports article publishing charges was provided by Universidad Científica del Sur Liliana Cruz-Ausejo reports a relationship with SUniversidad Científica del Sur, Facultad de Ciencias de la Salud that includes: funding grants.
